# CLUSTER ANALYSIS OF ATTENTIONAL PERFORMANCE AND BEHAVIORAL EXPRESSIONS IN ADULTS WITH ADHD SYMPTOMS

**DOI:** 10.1192/j.eurpsy.2023.2087

**Published:** 2023-07-19

**Authors:** L. R. R. Carreiro, I. T. Paes, M. M. M. Silva, A. G. Seabra

**Affiliations:** 1 Postgraduate Program in Developmental Disorders, Mackenzie Presbyterian University, São Paulo, Brazil

## Abstract

**Introduction:**

Attention Deficit Hyperactivity Disorder (ADHD) is characterized by a persistent pattern of inattention, hyperactivity, and impulsivity, which begins in childhood and often persists into adulthood. ADHD has a heterogeneous expression with diversity in behavioral symptoms, cognitive deficits, and comorbidities. So, it is possible to consider it a spectrum with different losses.

**Objectives:**

To describe clusters of multiple neuropsychological, attentional, and behavioral measures in adults with symptoms of ADHD. It could help to seek new directions to examine heterogeneity from a dimensional approach to ADHD.

**Methods:**

120 adults between 18 and 52 years old (m= 29.5) with ADHD symptoms participated in this study. Performance indices on computerized neuropsychological tests of attention (voluntary, automatic, temporal, and sustained), behavioral self-report scales for ADHD (ASRS-18), impulsivity (BIS-11) executive dysfunction (BDEFS), and functionality, emotional and behavioral problems (Adult Self-Report - ASR of ASEBA) were analyzed. Cluster analysis processed the data to find subgroups based on the scores of instruments. The NbClust tested the best number of clusters that converge to a solution.

**Results:**

The 3 clusters solution was obtained by comparing Z scores for each indicator. In cluster 1, the ADHD symptoms were equivalent but expressed more hyperactivity than in other clusters. Also, higher levels of functional impairments and executive dysfunctions (motivation, emotional regulation, and anxiety/depression) were identified. In the attentional neuropsychological tasks, the indices express a lower level. Cluster 2 expressed a higher level of inattention and attentional, motor, and non-planning impulsivity, and functional impairments in the academic, professional, and legal risk areas. Cluster 3 was the subgroup with the lowest level of symptoms of ADHD.

**Conclusions:**

This study identified differences in performances that contribute to understanding the cognitive, behavioral, and emotional expressions of ADHD. Three groups of different prejudices levels should be considered in the development of evaluative models in new studies to consider the spectrum of ADHD.

Financial support: FAPESP [grant 2019/20757-5, 2019/21773-4, 2020/14800-2]; CAPES Proex [grant 0426/2021, 23038.006837/2021-73]; Mackpesquisa; CNPq [grant 307443/2019-1]

**Disclosure of Interest:**

None Declared

